# Model-Based Conditional Weighted Residuals Analysis for Structural Model Assessment

**DOI:** 10.1208/s12248-019-0305-2

**Published:** 2019-02-27

**Authors:** Moustafa M. A. Ibrahim, Sebastian Ueckert, Svetlana Freiberga, Maria C. Kjellsson, Mats O. Karlsson

**Affiliations:** 1 Department of Pharmaceutical Biosciences, Uppsala University, Uppsala, Sweden; 2 Department of Pharmacy Practice, Helwan University, Cairo, Egypt

**Keywords:** conditional weighted residuals, diagnostics, model evaluation, nonlinear mixed effects models, prediction bias, structural model

## Abstract

Nonlinear mixed effects models are widely used to describe longitudinal data to improve the efficiency of drug development process or increase the understanding of the studied disease. In such settings, the appropriateness of the modeling assumptions is critical in order to draw correct conclusions and must be carefully assessed for any substantial violations. Here, we propose a new method for structure model assessment, based on assessment of bias in conditional weighted residuals (CWRES). We illustrate this method by assessing prediction bias in two integrated models for glucose homeostasis, the integrated glucose-insulin (IGI) model, and the integrated minimal model (IMM). One dataset was simulated from each model then analyzed with the two models. CWRES outputted from each model fitting were modeled to capture systematic trends in CWRES as well as the magnitude of structural model misspecifications in terms of difference in objective function values (ΔOFV_Bias_). The estimates of CWRES bias were used to calculate the corresponding bias in conditional predictions by the inversion of first-order conditional estimation method’s covariance equation. Time, glucose, and insulin concentration predictions were the investigated independent variables. The new method identified correctly the bias in glucose sub-model of the integrated minimal model (IMM), when this bias occurred, and calculated the absolute and proportional magnitude of the resulting bias. CWRES bias versus the independent variables agreed well with the true trends of misspecification. This method is fast easily automated diagnostic tool for model development/evaluation process, and it is already implemented as part of the Perl-speaks-NONMEM software.

**Electronic supplementary material:**

The online version of this article (10.1208/s12248-019-0305-2) contains supplementary material, which is available to authorized users.

## INTRODUCTION

Nonlinear mixed effects (NLME) models are currently advocated to maximize the utilization of gained information throughout all the phases of drug development. These models are adopted for reducing sample size, calculating study power, confirming drug effects, selecting doses, and optimizing trial design as well as supporting final/interim analysis decisions (1). In such settings, the appropriateness of the modeling assumptions is critical in order to draw correct conclusions and the assumptions must be carefully assessed for any substantial violations. Usually, modeling assumptions are assessed from the available knowledge on physiological processes that are to be modeled. However, model misspecifications can occur when the incompatibility of a modeling assumption with the underlying system goes undetected/untested, even though the model appears to give an accurate description of the data (2).

Different numerical and visual techniques had been proposed as reliable model evaluation methods. Numerical diagnostics include assessment of parameters uncertainty, conditional weighted residuals (CWRES) (3), normalized prediction distribution errors (NPDE) (4), posterior predictive checks (PPC) (5), and numerical predictive checks (6). Visual diagnostics based on model predictions include scatterplots of observed versus predicted and residuals versus predicted, while simulation-based diagnostics include visual predictive checks (7) and the graphical versions of NPDE and PPC. The pros and cons of these techniques had been thoroughly discussed by Nguyen *et al.* (8) where it was clear that even though graphical tools can signal where the model fails to describe the data, none of them can quantify this model misspecification or the gain in goodness of fit upon correction.

Lately, a new diagnostic tool based on residual modeling has been proposed as an easy and fast automated tool for model development/evaluation process (9). Residual modeling showed the superiority of CWRES over other residuals, where CWRES modeling provided guidance for where a potential model misspecification occurred, similar to other visual diagnostics. In addition, it uniquely identified the nature and quantified the magnitude of this misspecifications in terms of objective function value (OFV). In this work, we present a new method based on CWRES modeling to assess structural assumptions as prediction bias in NLME models developed for continuous data, by back-extrapolating a CWRES-based bias using the first-order conditional estimation (FOCE) approximation. First, we introduce CWRES bias calculation, then we derive predication-bias correction based on the calculated CWRES bias. Afterwards, we illustrate the practical use of this method by assessing prediction bias in two integrated NLME models for glucose homeostasis, the integrated glucose-insulin (IGI) model and the integrated minimal model (IMM) (10,11). Both models consist of glucose and insulin sub-models with interconnecting control mechanisms, and were proposed to describe simultaneously the glucose-insulin regulation system following intravenous glucose tolerance test (IVGTT) in healthy subjects.

## METHODS

### Calculating CWRES Bias

CWRES data outputted from the NLME model execution was treated as the dependent variable (DV) and modeled first by a base model to estimate CWRES distribution mean and variance.

1 $$ {\overset{\rightharpoonup }{y}}_i={\Theta}_1+{\eta}_i+{\overset{\rightharpoonup }{\varepsilon}}_i $$

where ${\overset{\rightharpoonup }{y}}_i$ is a vector of CWRES data from individual *i*, Θ_1_ is the mean of CWRES, *η*_*i*_ is the random unexplained deviation of individual *i* from the typical value, with variance Ω, and ${\overset{\rightharpoonup }{\varepsilon}}_i$ is the vector of residual unexplained variability of individual *i*, with variance Σ and it is assumed to be independent identically distributed. The expected values of Θ_1_, Ω, and Σ are 0, 0, and 1, respectively, as CWRES are theoretically expected to follow a normal distribution with mean 0 and variance 1 for a correct model (3). This base model (Eq. 1) was then extended to estimate different means for *N* number of bins of the independent variable (IDV) at *N* − 1 cutoff points (X_1_,…, X_*N* − 1_) dictated by data density as follow:

2 $$ \mathrm{If}\ \left(\min \left(\mathrm{IDV}\right)<\mathrm{IDV}<{\mathrm{X}}_1\right)\kern2em {\overset{\rightharpoonup }{y}}_i={\Theta}_1+{\eta}_i+{\overset{\rightharpoonup }{\upvarepsilon}}_i $$

3 $$ \mathrm{If}\ \left({\mathrm{X}}_1<\mathrm{IDV}<{\mathrm{X}}_2\right)\kern5.5em {\overset{\rightharpoonup }{y}}_i={\Theta}_2+{\eta}_i+{\overset{\rightharpoonup }{\upvarepsilon}}_i $$

4 $$ \mathrm{If}\ \left({\mathrm{X}}_{N-1}<\mathrm{IDV}<\max \left(\mathrm{IDV}\right)\right)\kern2em {\overset{\rightharpoonup }{y}}_i={\Theta}_N+{\eta}_i+{\overset{\rightharpoonup }{\upvarepsilon}}_i $$

This captured systematic trends in CWRES as well as the magnitude of structural model misspecifications, measured by the difference in objective function values ΔOFV_Bias_ between base model objective function value OFV_Base_ and the extended model objective function value OFV_Extended_.

5 $$ {\Delta \mathrm{OFV}}_{\mathrm{Bias}}={\mathrm{OFV}}_{\mathrm{Extended}}-{\mathrm{OFV}}_{\mathrm{Base}} $$

The estimates of the bin specific means (Θ_1_,…, Θ_*N*_) are CWRES bias vector (*b*) of length *N*. Another vector $\overset{`}{b}$ is derived by extending *b* to have the same dimensions as ${\overset{\rightharpoonup }{y}}_i$ by repeating each bin specific mean for all observations within this IDV bin. Afterwards,$\overset{`}{b}$ is used to correct bias in conditional predictions by the inversion of FOCE covariance calculation as follow.

### Prediction-Bias Correction

Let *y*_*i*_ be the vector of observation for subject *i*, *E*(*Y*_*i*_) and *COV*(*Y*_*i*_) denote the expectation and the covariance-variance of the conditional predictions *Y*_*i*_ calculated by FOCE under the NLME model with CWRES (*r*):

6 $$ E\left({Y}_i\right)=f\left(\overset{\rightharpoonup }{\theta },{\widehat{\eta}}_i\right)-{\left.\frac{df}{d{\overset{\rightharpoonup }{\eta}}_i}\right|}_{{\overset{\rightharpoonup }{\eta}}_i={\widehat{\eta}}_i}\cdotp {\widehat{\eta}}_i $$

7 $$ COV\left({Y}_i\right)={\left.\frac{df}{d{\overset{\rightharpoonup }{\eta}}_i}\right|}_{{\overset{\rightharpoonup }{\eta}}_i={\widehat{\eta}}_i}\cdotp \Omega \cdotp {\left.\frac{d\overset{`}{f}}{d{\overset{\rightharpoonup }{\eta}}_i}\right|}_{{\overset{\rightharpoonup }{\eta}}_i={\widehat{\eta}}_i}+\mathit{\operatorname{diag}}\left(\kern0.5em {\left.\frac{dh}{d{\overset{\rightharpoonup }{\varepsilon}}_i}\right|}_{{\overset{\rightharpoonup }{\varepsilon}}_i=0}\cdotp \varSigma \cdotp {\left.\frac{d\overset{`}{h}}{d{\overset{\rightharpoonup }{\varepsilon}}_i}\right|}_{{\overset{\rightharpoonup }{\varepsilon}}_i=0}\right) $$

8 $$ r\left({Y}_i,{y}_i\right)= COV{\left({Y}_i\right)}^{-\frac{1}{2}}\left({y}_i-E\left({Y}_i\right)\right) $$

where *f* denotes individual model predictions, in which $\overset{\rightharpoonup }{\theta }$ is the vector of population fixed effects, ${\overset{\rightharpoonup }{\eta}}_i$ is vector of random unexplained individual deviation from the population fixed effects. *h* is the unexplained residual variability model, ${\overset{\rightharpoonup }{\varepsilon}}_i$ is the vector of residual errors, and ${\widehat{\eta}}_i$ is the vector of empirical Bayes estimates. Both random effects, ${\overset{\rightharpoonup }{\eta}}_i$ and${\overset{\rightharpoonup }{\varepsilon}}_i$, are assumed to follow normal distribution with mean 0 and covariance matrix Ω and Σ, respectively. For conditional predictions from the true model ${Y}_i^{\ast }$:

9 $$ E\left(r\left({Y}_i^{\ast },{y}_i\right)\right)=0\kern0.75em COV\left(r\left({Y}_i^{\ast },{y}_i\right)\right)=1 $$

Let ${Y}_i^{-}$ be conditional predictions from a misspecified model with biased CWRES $\overset{`}{b}$ :

10 $$ E\left(r\left({Y}_i^{-},{y}_i\right)\right)=\overset{`}{b} $$

By defining the distance ${\delta}_i={Y}_i^{-}-{Y}_i^{\ast }$, we get:

11 $$ E\left({Y}_i^{-}\right)=E\left({Y}_i^{\ast}\right)+{\delta}_i $$

12 $$ E\left(r\left(E\left({Y}_i^{\ast}\right)+{\delta}_i,{y}_i\right)\right)=\overset{`}{b} $$

13 $$ E\left( COV{\left({Y}_i^{\ast}\right)}^{-\frac{1}{2}}\left({y}_i-E\left({Y}_i^{\ast}\right)-{\delta}_i\right)\right)=\overset{`}{b} $$

14 $$ E\left({y}_i-E\left({Y}_i^{\ast}\right)-{\delta}_i\right)= COV{\left({Y}_i^{\ast}\right)}^{\frac{1}{2}}\bullet \overset{`}{b} $$

15 $$ E\left({y}_i\right)-E\left({Y}_i^{\ast}\right)-E\left({\delta}_i\right)= COV{\left({Y}_i^{\ast}\right)}^{\frac{1}{2}}\bullet \overset{`}{b} $$

16 $$ 0-{\delta}_i= COV{\left({Y}_i^{\ast}\right)}^{\frac{1}{2}}\bullet \overset{`}{b} $$

17 $$ {\delta}_i=- COV{\left({Y}_i^{-}\right)}^{\frac{1}{2}}\bullet \overset{`}{b} $$

Assuming $COV\left({Y}_i^{-}\right)= COV\left({Y}_i^{\ast}\right)$, then conditional predictions from misspecified model ${Y}_i^{-}$ can be corrected by *δ*_*i*_. The last assumption implies that $\overset{`}{b}$ explains all of the structural model misspecifications. The distance *δ*_*i*_ and the percentage change of conditional predictions ($\%\frac{\delta_i}{Y_i}$) can further be binned by same *N* bins of the IDV and averaged over all subjects to get vectors *δ* and % *δ*, respectively, both of *N* length for graphical purposes.

### NLME Models

We chose to demonstrate this method by assessing prediction bias in two integrated NLME models for glucose homeostasis, the IGI model and the IMM; simpler example can be found in the Supplementary materials. Both the IGI model and the IMM claimed an underlying physiologically plausible structure to explain glucose-insulin dynamic interaction while retaining parsimony. The IGI model, shown in Fig. I, was developed for both healthy subjects and patients with type 2 diabetes following labeled IVGTT, thus observations, i.e., dependent variables, included glucose, radiolabeled glucose (tracer), and insulin measurements. The glucose sub-model is a two-compartment model with a central compartment elimination that is divided into insulin-dependent clearance and insulin-independent clearance. The glucose sub-model has two effect compartments accounting for the control mechanisms of glucose on its own production and on second-phase insulin secretion, respectively. The insulin sub-model is a one compartment disposition model with one effect compartment for the effect of insulin on the regulation of glucose clearance. Upon glucose administration, insulin first-phase amount enters insulin first-phase compartment as a system response, then it is released into the insulin central compartment. The IGI model has been widely used in diabetes modeling with applications in exploring drug effects (12), disease progression (13), designing early clinical trials (14), and optimizing IVGTT design (15).

**Fig. I:**
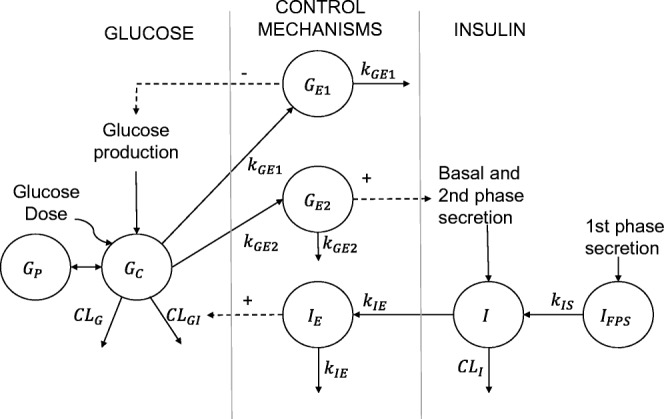
Schematic presentation of the integrated glucose-insulin model during IVGTT in healthy subjects. *G*_*C*_ and *G*_*P*_, central and peripheral compartments of glucose; *G*_*E*1_ and *G*_*E*2_, effect compartments of glucose on endogenous glucose production and second-phase insulin secretion, respectively; *I*, central compartment of insulin; *I*_*E*_, effect compartment of insulin on glucose; *I*_*FPS*_, insulin first phase compartment; *k*_*GE*1_, *k*_*GE*2_, *k*_*IE*_, and *k*_*IS*_, first order rate parameters; *CL*_*G*_, *CL*_*GI*_, and *CL*_*I*_, insulin-independent glucose clearance, insulin-dependent glucose clearance, and insulin clearance, respectively

The IMM was developed in healthy subjects following unlabeled insulin-modified IVGTT, so its data was lacking the unique information provided by radiolabeled glucose. The model is divided into two sub-models, glucose and insulin, based on the two-compartment glucose minimal model (16) and insulin minimal model (17), respectively (Fig. II). The glucose sub-model is a two-compartment model with elimination from the central compartment. Transit compartments are used to describe glucose kinetics in the first minutes after glucose dosing. The rate of change of glucose amount in central compartment $\dot{G_1}(t)$ is the difference between the rate of hepatic glucose production, the rate of glucose disappearance by liver uptake, the rate of glucose disappearance by peripheral tissue, and the distribution between central and peripheral compartments. Since unlabeled IVGTT data did not allow the explicit description of hepatic glucose production, hepatic glucose production and hepatic glucose uptake were lumped into a net hepatic glucose balance, leading to:

18 $$ \dot{G_1}={S}_G\cdotp {G}_b-\left({S}_G+X\left(\mathrm{t}\right)+{k}_{21}\right)\cdotp {G}_1\left(\mathrm{t}\right)+{k}_{12}\cdotp {G}_2\left(\mathrm{t}\right)\kern4em {G}_1(0)={G}_b $$

where *G*_1_(*t*), *G*_2_(*t*), and *G*_*b*_ are glucose amounts in central compartment, in peripheral compartment, and basal glucose amount, respectively; *k*_21_ and *k*_12_ are transfer rate parameters; *S*_*G*_ is glucose effectiveness, quantifying the ability of glucose to enhance its own rate of disappearance at basal insulin concentration and is the sum of two parameters: *k*_5_ that describes hepatic glucose uptake as well as the inhibitory effect of glucose on hepatic glucose production, and *k*_1_ that describes peripheral uptake as a function of glucose amount in central compartment; *X*(t) is the effect of insulin on glucose kinetics. The insulin sub-model consists of a two-compartment disposition model with elimination from the central compartment. A transit compartment was used to describe insulin first-phase secretion, while second-phase insulin secretion rate is derived proportional to glucose concentration. When insulin concentration in the central compartment is higher than its basal steady state concentration, it moves to a remote compartment, representing receptor pool for insulin binding to its target tissues, where it produces its effects to lower glucose concentration. The IMM was proposed to overcome the limitations of the traditional minimal models, while still deriving the important physiological indices: glucose effectiveness *S*_*G*_ and insulin sensitivity *S*_*I*_, for clinical diagnosis with estimates that are compatible with the traditional minimal model approach.

**Fig. II:**
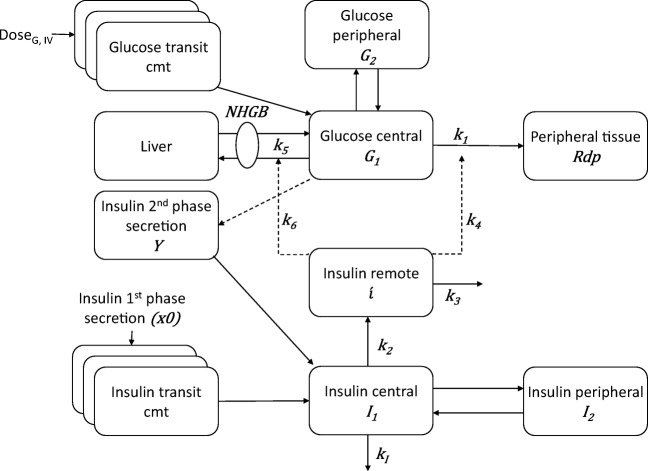
Schematic presentation of the integrated minimal model during IVGTT in healthy subjects. *G*_1_ and *G*_2_, central and peripheral compartments of glucose; *I*_1_ and *I*_2_, central and peripheral compartments of insulin; $\overset{\acute{\mkern6mu}}{i}$ insulin concentrations in the remote compartment; *Rdp*, rate of glucose disappearance by peripheral tissue uptake; *x*0, first phase insulin concentrations; *Y*, second-phase insulin secretion; *NHGB*, net hepatic glucose balance; *k*_1_ and *k*_5_, glucose model parameters; *k*_2_, *k*_3_, *k*_4_, and *k*_6_ parameters of insulin action; *k*_*I*_, insulin elimination rate constant

### Settings

One dataset was simulated from each of the IGI model and the IMM according to a standard IVGTT protocol: 0.33 g/kg bolus of glucose with blood sampling at 0, 2, 3, 4, 5, 6, 8, 10, 12, 15, 18, 20, 22, 24, 26, 28, 30, 35, 40, 45, 50, 55, 60, 70, 80, 100,120,140,160,180, 210, and 240 min. Each simulated data set was analyzed with the two models, and visual predictive checks were performed to investigate the goodness of each fit. CWRES outputted from each model fitting was separated based on the two DVs glucose and insulin, where after CWRES for each DV was modeled to calculate ΔOFV_Bias_, *b*, and *δ*_*i*_ as shown in Fig. III. Time, glucose population predictions (PRED), and insulin PRED were the investigated IDV by separate estimations. To evaluate the performance of our method, we calculated the % known bias in conditional predictions of each DV to be the reference bias estimates in *Eq*. 19, where *Y*_*i*, *sim*_ is the simulated conditional predictions and *Y*_*i*, *est*_ is the estimated conditional predictions of this DV.

19 $$ \% known\ bias=100\bullet \frac{Y_{i, sim}-{Y}_{i, est}}{Y_{i, sim}} $$

**Fig. III:**
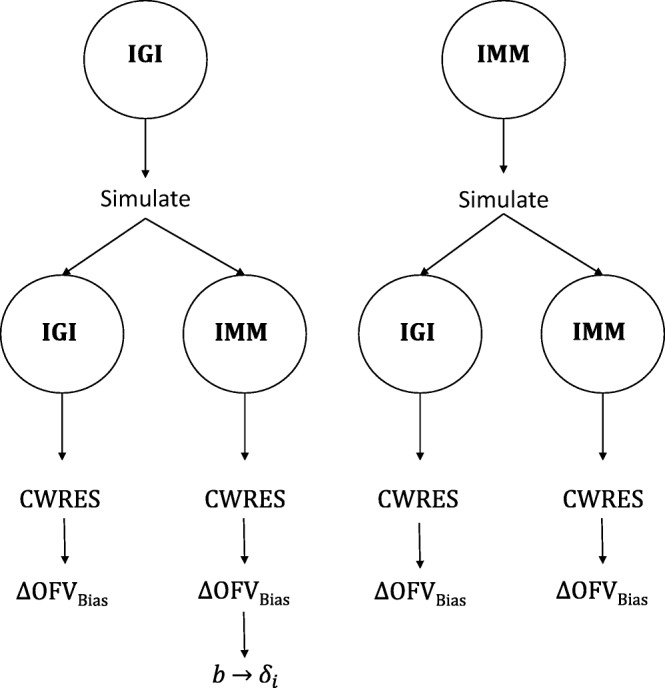
One dataset was simulated from each model according to a standard IVGTT protocol, then analyzed by the two models. CWRES for each DV from each model fitting was further analyzed to calculate ΔOFV_Bias_, *b*, and *δ*_*i*_

Also, to avoid bias introduced by binning, a previously recommended random binning technique (18) was implemented by the following specifications, with number of bins being *N* and the minimum number of observations per bin being *M*:

Step 1: Sort CWRES data by the selected IDV.
Step 2: Generate *N* − 1 bin boundaries randomly, based on the IDV.
Step 3: Group CWRES data based on generated bin boundaries.
Step 4: Estimate *b* → *δ*_*i*_.
Step 5: Repeat steps (2–4) 500 times.

In our investigations, using time as IDV, *N* was set to 10, otherwise *N* was set to 5 and *M* was set to 25. Nonlinear mixed effects analysis, statistical and graphical assessment was performed in PSN (19), NONMEM version 7.3 (20) and R (21). Simulated conditional predictions *Y*_*i*, *sim*_ was outputted from NONMEM using $ETAS and $ESTIMATION with options MCETA = 1 FNLETA = 2.

## RESULTS

When either of the two data sets was analyzed with the IGI model or data simulated by the IMM was analyzed by the IMM, ΔOFV_Bias_ was non-significant for both DVs (glucose and insulin) at ${\mathcal{X}}_{0.05}^2$(10 degree of freedom) when time was the IDV, and at ${\mathcal{X}}_{0.05}^2$(5) when glucose PRED or insulin PRED was the IDV. When data simulated by the IGI was analyzed with the IMM, ΔOFV_Bias_ was significant for glucose versus the three IDVs, but not for insulin as shown in Table I.

**Table I:** Calculated ΔOFV_Bias_ for the Two Dependent Variables Glucose and Insulin for the IGI Model and the IMM Versus the Three Investigated Independent Variables: Time, Glucose PRED, and Insulin PRED. Significant Bias is Indicated in the Table with Italics

		ΔOFV_Bias_					
		Glucose			Insulin		
Simulation	Estimation	Time	Glucose PRED	Insulin PRED	Time	Glucose PRED	Insulin PRED
IGI	IGI	13.64	6.57	6.26	5.56	2.99	3.77
	IMM	*50.15*	*15.38*	*13.83*	13.07	4.02	3.48
IMM	IGI	6.67	3.66	1.23	10.95	6.48	6.06
	IMM	13.14	6.43	8.58	2.92	3.34	3.69

The first two rows of the table contains ΔOFV_Bias_ when simulating with the IGI model and estimating with both the IGI model and the IMM versus time, glucose PRED and insulin PRED, while the second two rows of the table contains ΔOFV_Bias_when simulating with the IMM and estimating with the two models versus the same IDVs

Plots of estimated bias in conditional predictions calculated by CWRES modeling % *δ* versus the three investigated IDVs are shown in Figs. IV, V, and VII, where an over prediction bias in glucose sub-model is evident using both fixed or random binning. Visual predictive checks of the IMM when fitted to data simulated from the IGI model are shown in Fig. VII, only glucose sub-model showed an over prediction where the 95% confidence interval around the median of the simulations from the IMM is higher than the median of the data simulated from the IGI model, similar to where this over prediction was captured by the new method. In addition, the new method showed the bias against the interacting predictions of glucose and insulin, which is not routinely checked with visual predictive checks. The over prediction in the IMM glucose sub-model was found at early time points (< 150 min) with binning based on time, at high glucose concentrations (> 90 mg/dl) with binning based on glucose PRED, and at almost all bins with binning based on insulin PRED. The absolute and proportional magnitude of the over prediction versus the three IDVs showed a good agreement between the estimates calculated based on CWRES modeling (% *δ*) and the reference estimates (% known bias), as presented in Table II and shown in Figs. IV, V, and VI. Finally, these results correctly pointed out a model misspecification in glucose sub-model of the IMM, similar to previously reported results with another analysis methods (22,23).

**Fig. VI:**
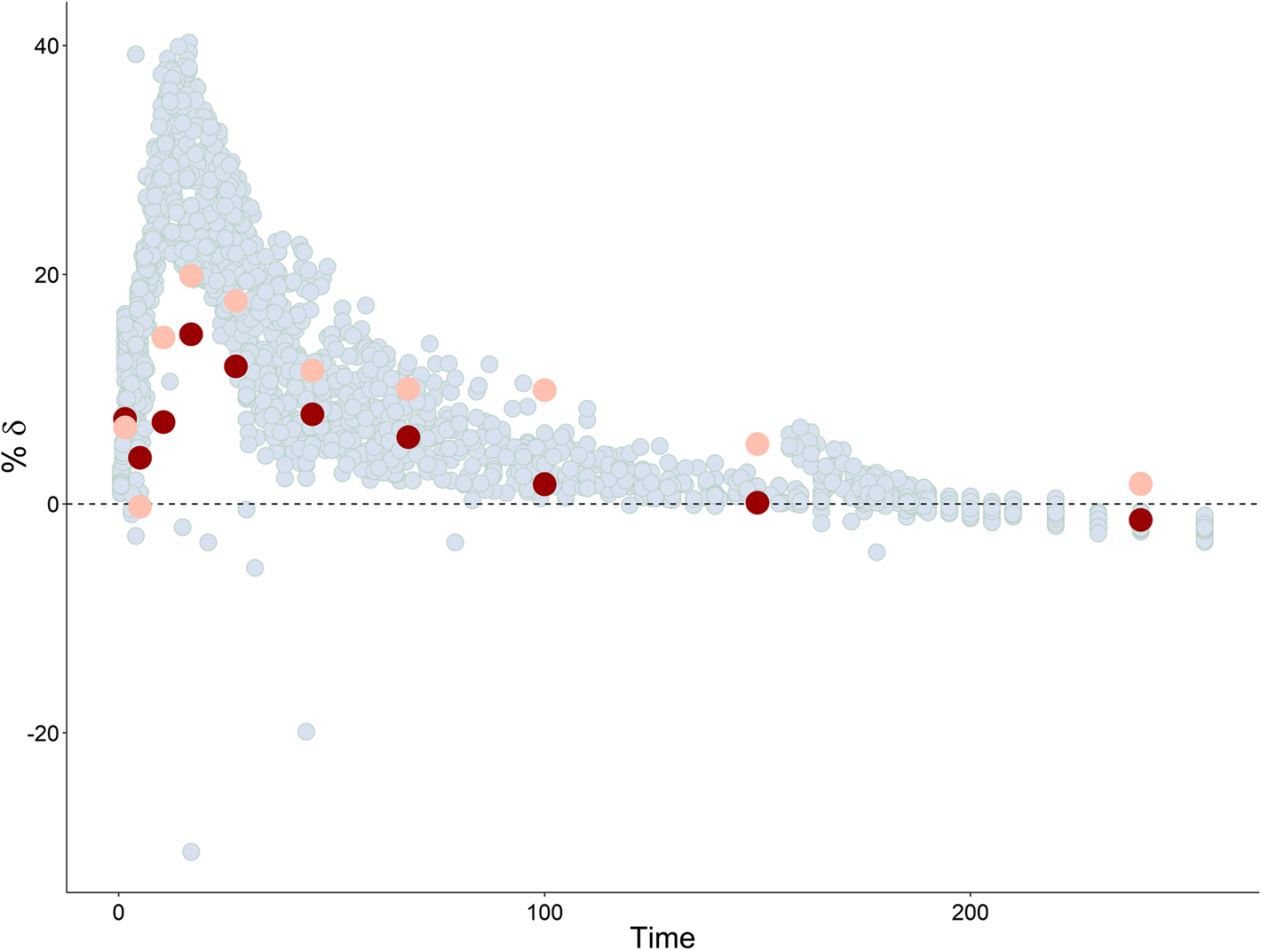
Plot of bias calculated in glucose sub-model by % *δ* (red), % known bias (rose), and random binning (ice blue) versus time, when the IMM was fitted to data simulated by the IGI model

**Fig. V:**
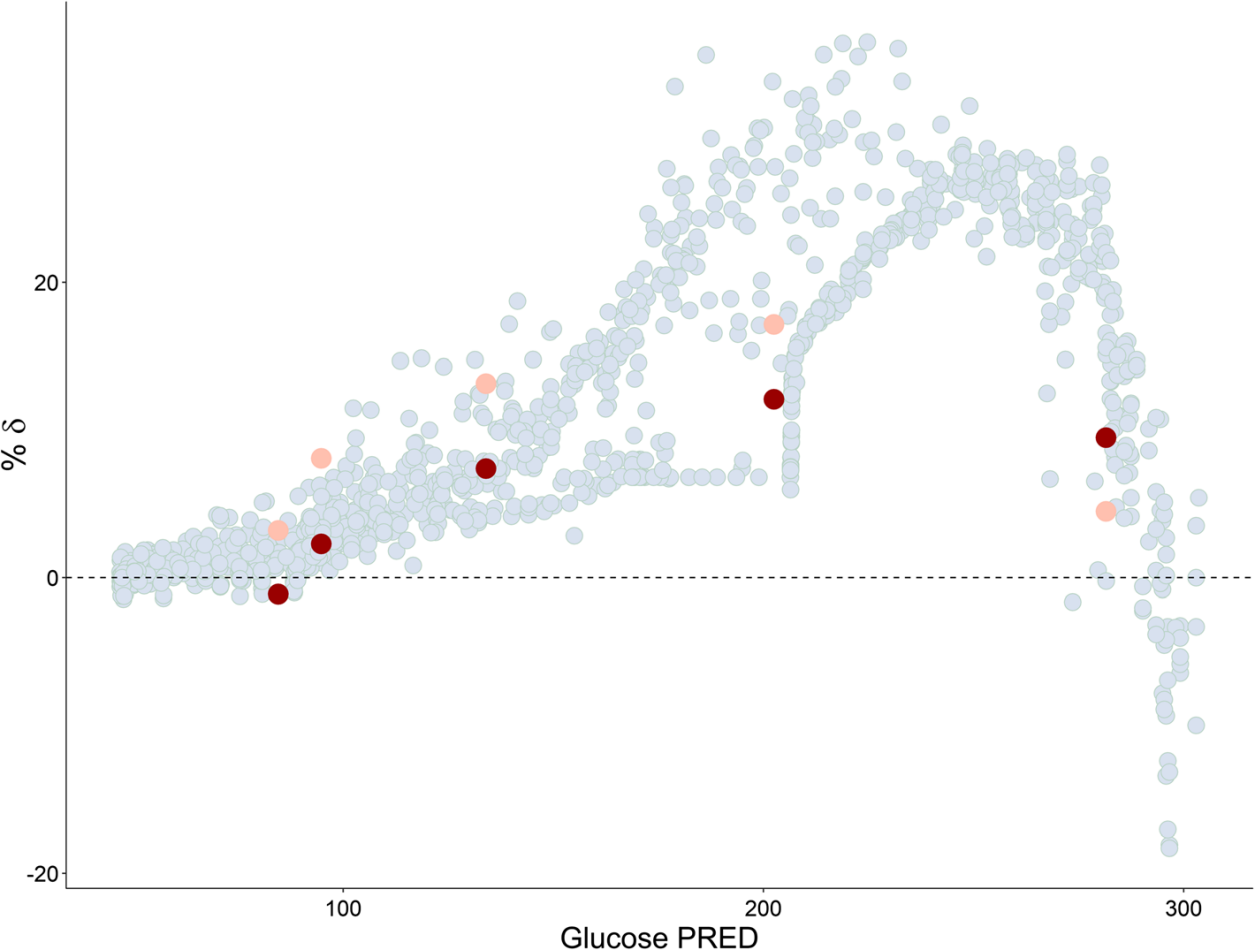
Plot of bias calculated in glucose sub-model by % *δ* (red), % known bias (rose), and random binning (ice blue) versus glucose PRED, when the IMM was fitted to data simulated by the IGI model

**Fig. VI:**
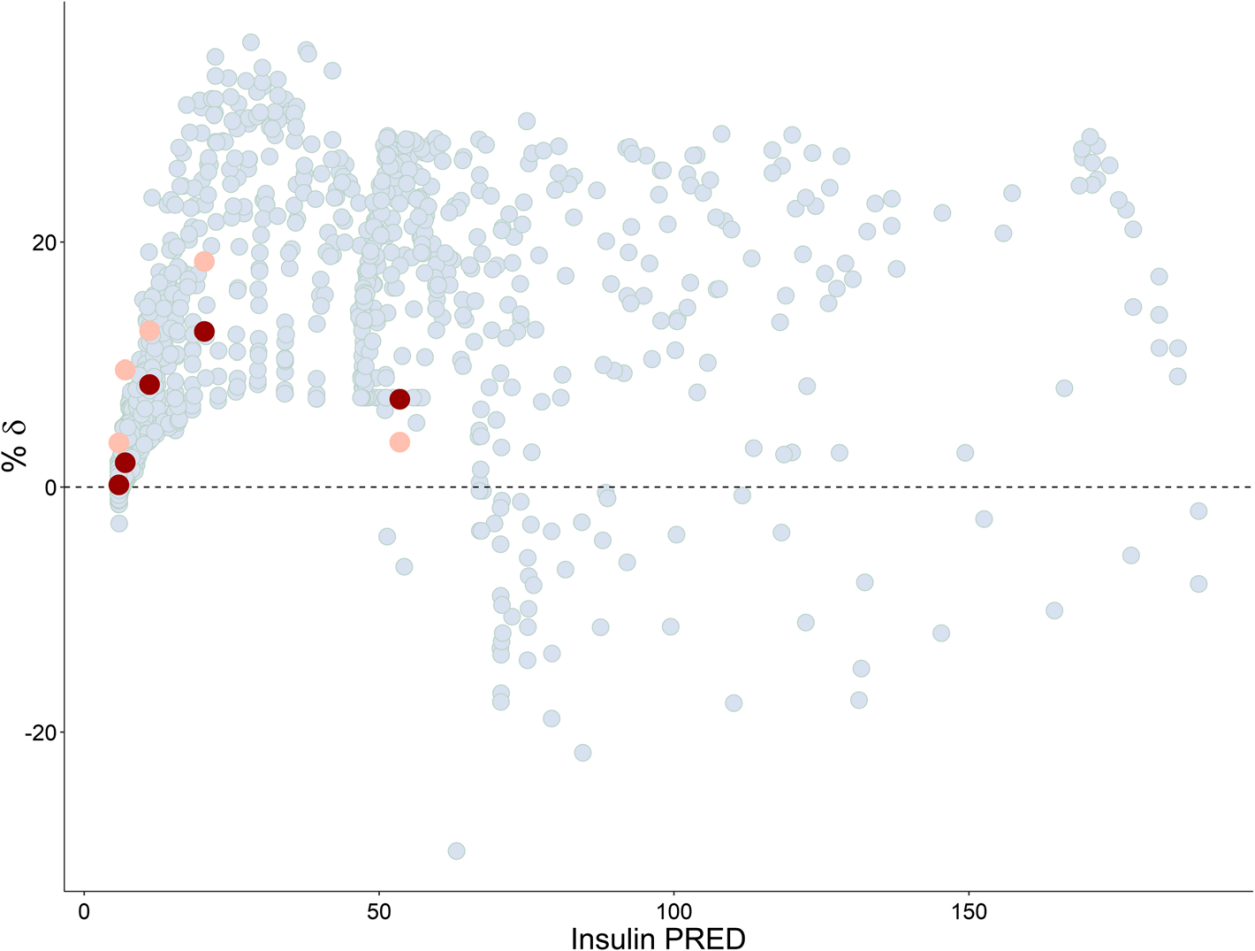
Plot of bias calculated in glucose sub-model by % *δ* (red), % known bias (rose), and random binning (ice blue) versus insulin PRED, when the IMM was fitted to data simulated by the IGI model

**Fig. VII:**
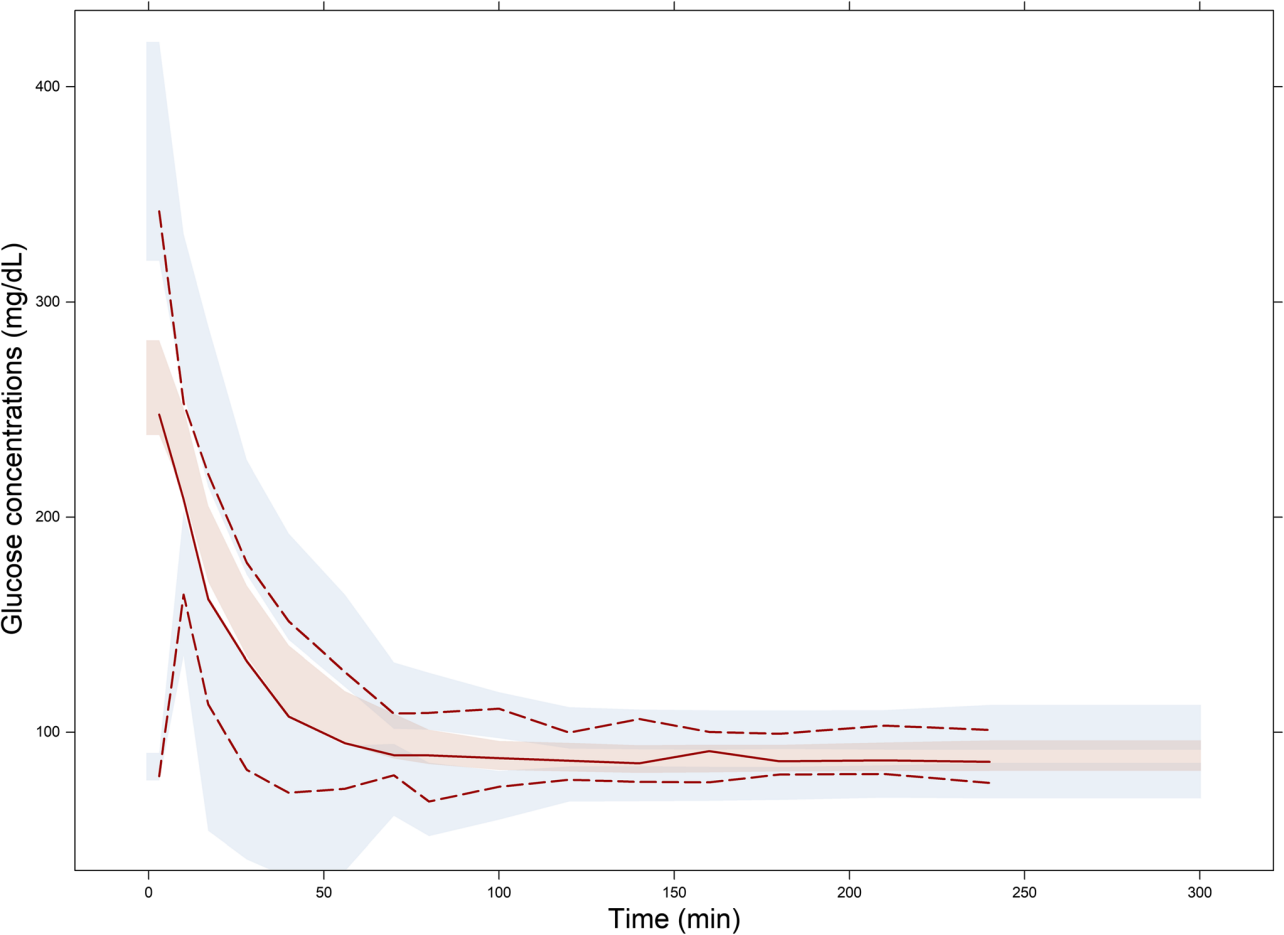
Visual predictive check VPC of total glucose concentrations, where 500 data sets were simulated from the IMM fitted to data simulated by the IGI model. The solid line is the median of data simulated by the IGI model, the dashed lines are the 5th and 95th percentiles of data simulated by the IGI model, and the shaded areas are the 95% confidence intervals of the simulations from the IMM around the same percentiles.

**Table II:** Estimates of *b*, % *δ*, and % Known Bias of the IMM Glucose Sub-model Versus the Three Investigated Independent Variables After Fitting the Model to Data Simulated from the IGI Model

Glucose								
Time			Glucose PRED			Insulin PRED		
*b*	% *δ*	% known bias	*b*	% *δ*	% known bias	*b*	% *δ*	% known bias
− 0.59	7.4	6.66	0.16	− 1.1	3.21	0.03	0.2	3.63
0.32	4.7	− 0.24	− 0.08	2.3	8.10	0.01	2	9.58
− 0.79	13.7	14.53	0.15	7.4	13.16	0.13	8.4	12.76
− 0.54	14.8	19.92	− 0.36	12.1	17.15	−0.43	12.7	18.43
− 0.01	12	17.72	− 0.21	9.5	4.5	−0.11	7.2	3.68
0.28	7.8	11.63						
− 0.12	5.8	10.03						
0.03	1.7	9.92						
0.04	0.1	5.20						
0.24	− 1.4	1.73						

## DISCUSSION

Nonlinear mixed effects modeling requires assumptions for handling different types of data and the different model components: structural, covariate, and stochastic models; since these assumptions are interconnected with each other such that a violation of one may have consequences for the apparent appropriateness of others, it becomes more challenging to correctly address such violation (8). One of the recently developed methods for model evaluation is CWRES post-processing. By parametric modeling of either the mean or the variance of CWRES distribution, it is possible to identify and quantify if a model misspecification is present and whether this model misspecification arises from the structural model or the stochastic model, in a fast and robust way (9). Based on CWRES modeling, we developed a new method to assess structural assumptions as prediction bias in NLME models developed for continuous data. The new method first calculated the bias in the mean of CWRES distribution, then the deviation between conditional predictions of a misspecified structural model, and expected true structural model, relying on the fact that CWRES under the true structure model is normally distributed with mean 0 and variance 1. We successfully applied the new method to two integrated complex models for glucose homeostasis, the IGI model, and the IMM. Both models claimed an underlying physiologically plausible structure, albeit different, to explain glucose-insulin dynamic interaction with the least possible number of estimated parameters, and so hypothetically both models are less prone to prediction bias. Our method correctly spotted the violation of the underlying structural model assumptions with the highest impact on the IMM performance, similar to % known bias and in agreement to previous investigations (22,23).

Both models use a two-compartment disposition model to describe glucose kinetics with elimination from central compartment. The elimination is divided into two pathways, defined differently in the two models. The IGI models assumes two pathways based on glucose transporters of the uptake tissue, either insulin sensitive transporters, e.g., GLUT4 or insulin insensitive transporters, e.g., GLUT2, while the IMM assumes two pathways based on the anatomy of uptake tissue, either peripheral or hepatic tissue, with each elimination further classified into insulin dependent or not. This difference in elimination as well as the absence of tracer data led to the IMM assumptions regarding net hepatic glucose balance and the hybrid nature of glucose and insulin effect parameters on hepatic tissue. Net hepatic balance is the difference between hepatic glucose production and hepatic glucose uptake, taking positive values when production is dominating and negative values if uptake is dominating and is mathematically derived as the difference between an extrapolated value of net hepatic balance at zero glucose concentrations and the hybrid effects of glucose and insulin to inhibit hepatic glucose production as well as enhancing hepatic glucose uptake (24). Hence, glucose effect parameter *S*_*G*_ is simultaneously measuring both mass flow and control mechanism through *k*_5_, its estimate is unrealistically large. This overestimation of glucose effect on glucose disappearance, constrained insulin effects *X*(*t*) on glucose disappearance to take low estimates, creating undesired compensation bias in the rest of glucose sub-model parameters (22). As insulin contributes to glucose elimination only at concentrations higher than basal insulin concentrations, and the hybrid parameter of glucose effects contributes to steady state conditions of glucose, both production and clearance, the impact of these biased parameters cancel out at steady state concentrations of glucose (~ 90 mg/dL). The impact of these biased parameters is magnified on system perturbation where insulin reaches effective concentrations in the remote compartment, but insulin dynamic effects in the model are constrained to underestimate the true consequences of these insulin effective concentrations on glucose disappearance curve. The impact of these biased parameters decreases again as insulin concentrations in remote compartment decrease toward insulin basal concentrations. This behavior explains the captured bias when simulating with the IGI model and estimating with the IMM model, as shown in Figs. 4, 5, and 6. Bias peaks immediately after first-phase insulin secretion then fades away with declining insulin concentrations. This happens at time points before 150 min, when glucose concentrations were higher than 90 mg/dL and when insulin concentrations were above basal insulin. Noting that insulin concentrations peak before glucose concentration, as lower bolus of similar rate of absorptions and central volumes of distributions, this may be the reason behind glucose concentrations higher than 280 mg/dL showed less bias than glucose concentrations in the range 200–280 mg/dL. Also, when simulating with the IMM and estimating with both models, ΔOFV_Bias_ was lower for the IGI model estimations, as presented in Table I, concluding that the IGI model structural assumptions regarding glucose kinetics were less prone to significant misspecifications. Finally, the magnitude of the IMM glucose sub-model bias peaked to 20% of conditional prediction of glucose, which is a considerably high percentage for such integrated system, and in light of a previous study (14), the utilization of such model in drug development to explore drug effects enhancing glucose disappearance will result in the misleading conclusions of overestimating drug effects on insulin-independent glucose clearance and underestimating drug effects on insulin-dependent glucose clearance.

Regarding insulin kinetics, the IGI model and the IMM assumed different disposition models, none showed significant ΔOFV_Bias_, and both models behaved ideally in a sense that when simulating with the IGI model and estimating with both models, ΔOFV_Bias_ was lower for the IGI model estimations, likewise when simulating with the IMM and estimating with both models, ΔOFV_Bias_ was lower for IMM estimations, as estimating and simulating with same model in absence of a high impact misspecification should always be almost bias free, unless the used estimation method is inappropriate. We also added a simple PK example as Supplementary material to explain and show the step-by-step implementation of our method in R.

Results from this new method should be interpreted within the context of two main factors and their impact on the purpose of the modeling exercise: the significance of ΔOFV_Bias_ and the magnitude of the detected bias % *δ*. For instance, if the purpose of the model was to physiologically describe an underlining system or derive physiological indices for clinical diagnosis as the IMM, then a high % *δ* in the dynamic relation between glucose and insulin must be addressed even if not accompanied with a significant ΔOFV_Bias_. Our new method is generalizable to all NLME models developed for continuous data and is independent of the used estimation method or analysis software if CWRES is available and calculated in the same way. This method inherits the unique merits of CWRES modeling, as being fast, robust, and not suffering from local minima problems. Noting that the method depends on the way of IDV binning. How and where to set the binning is subjective and up to modelers to choose; here we used data density, which was not supporting 10 bins for glucose PRED or insulin PRED as the IDV. Though being time consuming and computing intensive, random binning technique allowed horizontal exploration of additional bins that probably would not be subjectively selected, giving more insight of the present trends in CWRES distribution, and it provided vertical exploration for bins with higher probability of being selected, similar to confidence intervals. How to handle a detected bias is model and purpose dependent with no general recommendations; however, by using different IDVs in the visualization of the bias, clues on which part of the model that is misspecified can be revealed. When model predictions are too close to zero, it will not be possible to calculate %*δ*, and *δ* should be used instead.

Unlike residual post-processing (9), which can be applied to other residuals as NPDE and CWRESI, our method was derived only for CWRES as the last outperformed other residuals in residual error model identification. Different derivations will be needed for prediction-bias correction with other residuals and that was not explored in our work.

In conclusion, a new fast and easily automated diagnostic method for structural model assessment was successfully developed, evaluated, and applied to two integrated complex semi-mechanistic models. The new method can identify structural misspecification, wherever this misspecification occurs, and quantify its magnitude and impact on goodness of fit. This method is already implemented in PsN as part of qa tool (available from version 4.8.1) for model development/evaluation process.

## Electronic Supplementary Material

ESM 1 (DOCX 4.55 mb)

ESM 2 (ZIP 71.3 kb)
