# Supplementary material for: Model-Based Conditional Weighted Residuals Analysis for Structural Model Assessment
Source: AAPS J. 2019 Feb 27;21(3):34. doi: 10.1208/s12248-019-0305-2 (PMC6394649; doi:10.1208/s12248-019-0305-2)
Supplement: Supplementary file 1 — (DOCX 4.55 mb) [file 12248_2019_305_MOESM1_ESM.docx]

**Simple PK example**

In this example we used a two-compartment PK model with zero order absorption and Michaelis–Menten elimination to simulate a dataset of 100 subjects. The simulated data set was then used to fit two models: a true model (same as the simulation model) and a misspecified model that is the same as the simulation model except for using 1^st^ order absorption process instead of the true zero order absorption process. A plot of fitted model predictions from the misspecified model and the simulated model prediction from the true model is shown in **Figure 1**. Afterward CWRES data outputted from the true and misspecified models’ fits was modeled to calculate ${\Delta OFV}_{\mathrm{Bias}}$, $b$, and $\delta_{i}$. A plot of the percentage estimated bias in the misspecified model’s predictions by % $\delta$ (red) versus time is shown in **Figure 2**, capturing correctly the misspecifications as displayed by the good agreement between % $\delta$ and the % true bias (blue). The % true bias is calculated as the percentage difference between the simulated and estimated model predictions. ${\Delta OFV}_{\mathrm{Bias}}$ and the simulated parameters are reported in **Table 1**.


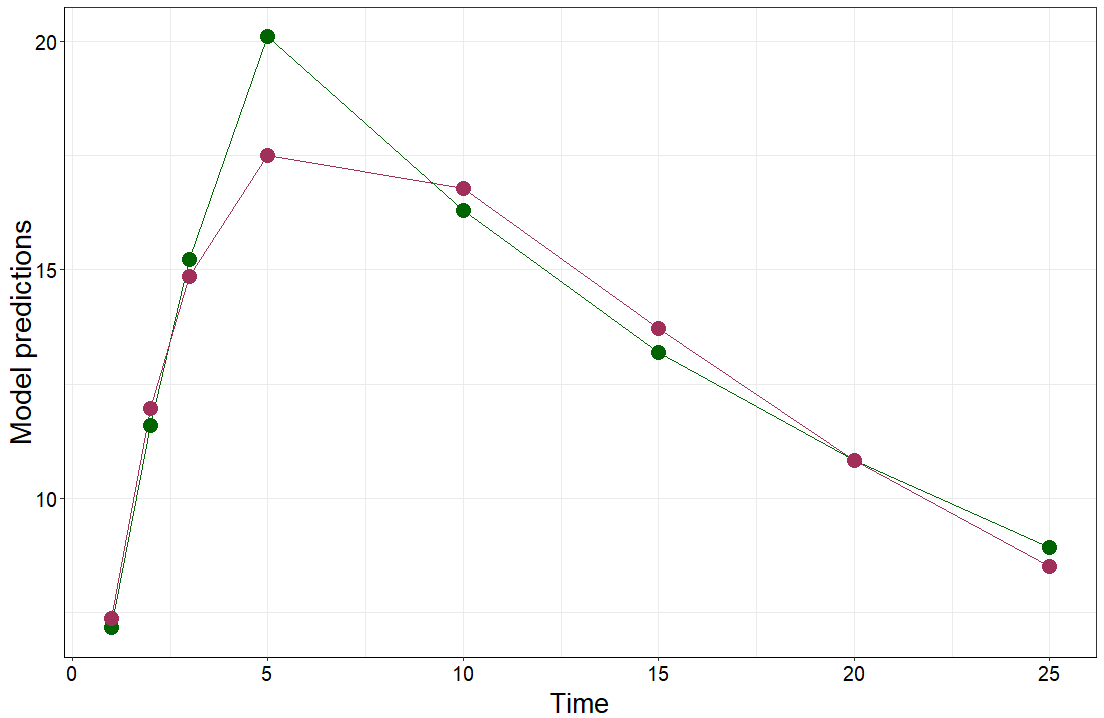


**Figure 1.** Plot of model predictions from the misspecified model (red) and the true model (green) versus time.


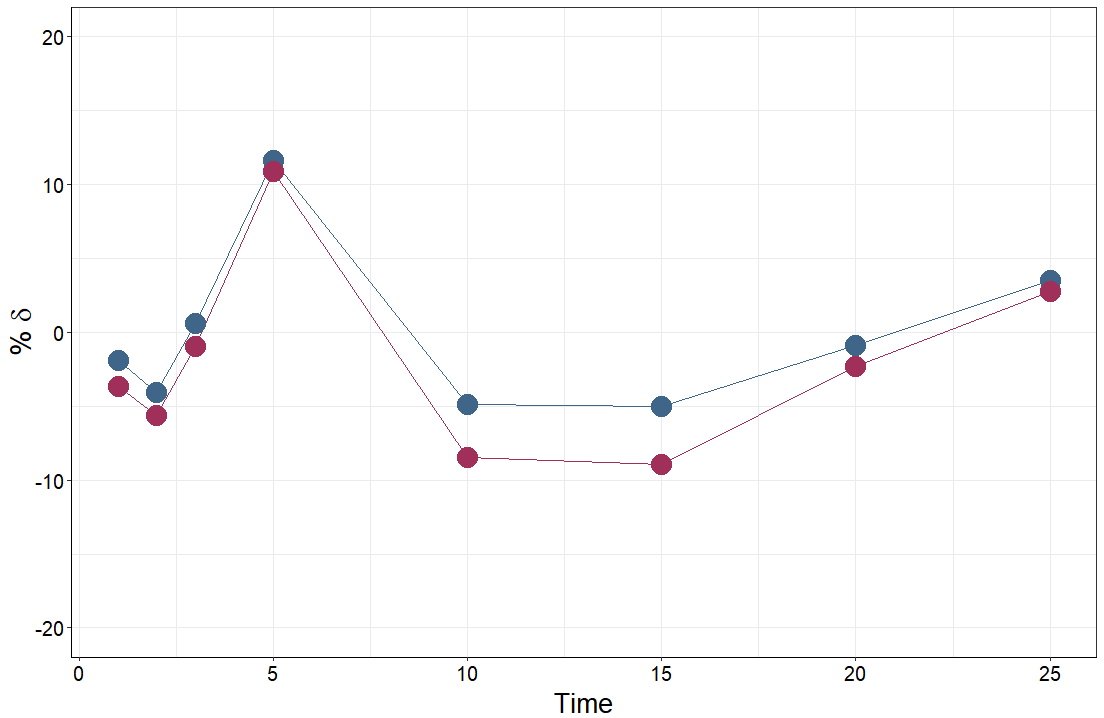


**Figure 2**. Plot of the bias calculated in the misspecified model by % $\delta$ (red) and % true bias (blue) versus time, when the misspecified model was fitted to data simulated by the true model.

**Table 1.** Simulation specifications and ${\Delta OFV}_{\mathrm{Bias}}$**.**

| True model | Misspecification | ${\Delta OFV}_{\mathrm{Bias}}$ | Simulated parameters | |
| --- | --- | --- | --- | --- |
| Zero order absorption | 1^st^ order absorption | -165.8 | $V_{c}$ | 4.14 |
|  | | | $V_{P}$ | 7 |
|  |  |  | ${ka}_{0}$ | 10.28 |
|  |  |  | $Q$ | 3.24 |
|  |  |  | $KM$ | 16.34 |
|  |  |  | $VMAX$ | 9.21 |

$V_{c}$ volume of central compartment, $V_{p}$ volume of peripheral compartment, ${ka}_{0}$ zero order absorption rate, $Q$ intercompartmental clearance, $KM$ and $VMAX$ Michaelis–Menten elimination parameters.
